# Radioiodinated Exendin-4 Is Superior to the Radiometal-Labelled Glucagon-Like Peptide-1 Receptor Probes Overcoming Their High Kidney Uptake

**DOI:** 10.1371/journal.pone.0170435

**Published:** 2017-01-19

**Authors:** Tilman Läppchen, Roswitha Tönnesmann, Jos Eersels, Philipp T. Meyer, Helmut R. Maecke, Svetlana N. Rylova

**Affiliations:** 1 Department of Nuclear Medicine, Medical Center–University of Freiburg, Faculty of Medicine, University of Freiburg, Germany; 2 Department of Nuclear Medicine, Inselspital, Bern University Hospital and University of Bern, Bern, Switzerland; 3 Department of Radiology and Nuclear Medicine, VU University Medical Centre, Amsterdam, The Netherlands; 4 German Cancer Consortium (DKTK), Heidelberg, Germany; 5 German Cancer Research Center (DKFZ), Heidelberg, Germany; Wayne State University, UNITED STATES

## Abstract

GLP-1 receptors are ideal targets for preoperative imaging of benign insulinoma and for quantifying the beta cell mass. The existing clinical tracers targeting GLP-1R are all agonists with low specific activity and very high kidney uptake. In order to solve those issues we evaluated GLP-1R agonist Ex-4 and antagonist Ex(9–39) radioiodinated at Tyr^40^ side by side with [Nle^14^,Lys^40^(Ahx-DOTA-^68^Ga)NH_2_]Ex-4 (^68^Ga-Ex-4) used in the clinic. The Kd, Bmax, internalization and binding kinetics of [Nle^14^,^125^I-Tyr^40^-NH_2_]Ex-4 and [Nle^14^,^125^I-Tyr^40^-NH_2_]Ex(9–39) were studied *in vitro* using Ins-1E cells. Biodistribution and imaging studies were performed in nude mice bearing Ins-1E xenografts. *In vitro* evaluation demonstrated high affinity binding of the [Nle^14^,^125^I-Tyr^40^-NH_2_]Ex-4 agonist to the Ins-1E cells with fast internalization kinetics reaching a plateau after 30 min. The antagonist [Nle^14^,^125^I-Tyr^40^-NH_2_]Ex(9–39) did not internalize and had a 4–fold higher K_d_ value compared to the agonist. In contrast to [Nle^14^,^125^I-Tyr^40^-NH_2_]Ex(9–39), which showed low and transient tumor uptake, [Nle^14^,^125^I-Tyr^40^-NH_2_]Ex-4 demonstrated excellent *in vivo* binding properties with tumor uptake identical to that of ^68^Ga-Ex-4, but substantially lower kidney uptake resulting in a tumor-to-kidney ratio of 9.7 at 1 h compared to 0.3 with ^68^Ga-Ex-4. Accumulation of activity in thyroid and stomach for both peptides, which was effectively blocked by irenat, confirms that *in vivo* deiodination is the mechanism behind the low kidney retention of iodinated peptides. The ^124^I congener of [Nle^14^,^125^I-Tyr^40^-NH_2_]Ex-4 demonstrated a similar favourable biodistribution profile in the PET imaging studies in contrast to the typical biodistribution pattern of [Nle^14^,Lys^40^(Ahx-DOTA-^68^Ga)NH_2_]Ex-4. Our results demonstrate that iodinated Ex-4 is a very promising tracer for imaging of benign insulinomas. It solves the problem of high kidney uptake of the radiometal-labelled tracers by improving the tumor-to-kidney ratio measured for [Nle^14^,Lys^40^(Ahx-DOTA-^68^Ga)NH_2_]Ex-4 by 32 fold.

## Introduction

Glucagon like peptide-1 receptors (GLP-1R) are ideal targets for the imaging of benign insulinoma due to their high density on the surface of more than 90% of these tumors [1]. Physiological expression of GLP-1 receptors on the pancreatic beta cells is also being exploited preclinically and potentially clinically for imaging of the pancreatic beta cell mass to quantify the loss of islets in type 1 diabetes [2,3]. Since islet transplantation may be a promising treatment option for patients with diabetes, the GLP-1 targeted imaging is being explored for visualization of transplanted islets and monitoring their survival [4,5].

In case of benign insulinoma, preoperative localization plays a crucial role in the successful outcome of the surgery and in sparing the healthy pancreatic tissue. However, due to the small size of insulinoma lesions (often less than 2 cm) the precise localization is very challenging using conventional imaging techniques having a sensitivity of only 47% [6]. This prompted an intensive research into developing more sensitive and non-invasive imaging modalities to localize benign insulinoma in the pancreas. A variety of GLP-1R targeting peptide probes labelled with ^111^In [7,8], ^99m^Tc [8], ^123^I [9] for SPECT and with ^68^Ga [3,8,10–13], ^64^Cu [3,14], ^18^F [5,15,16], and ^89^Zr [13] for PET imaging showed promising results in preclinical studies. Some of the tracers have been successfully translated into the clinic [17–22] and showed highest sensitivity among the available techniques in detecting ‘‘hidden” insulinomas in patients.

Despite the great clinical potential, several aspects of the existing GLP-1R specific tracers would greatly benefit from further improvements. Firstly, the high and persistent kidney uptake of radiometal-labelled tracers not only leads to an unnecessary high radiation dose to the kidney but also can complicate the preoperative and intraoperative insulinoma detection in the head and tail of the pancreas [17,20,23]. Secondly, the specific activity of the existing tracers is relatively low requiring higher peptide mass for adequate activity to achieve a good image quality. The agonist peptide dose is however limited due to the risk of insulin-related side effects. GLP-1 receptor agonists belong to the potent incretin hormones; after binding to the receptor they internalize and activate GLP-1 receptor signalling leading to the glucose-dependent insulin secretion.

For this reason, antagonist probes would be much more attractive because they would not activate the downstream signalling. Moreover, antagonist ligands for other G-protein coupled receptors (GPCRs), including gastrin-releasing peptide receptor and somatostatin receptors have been shown to target more binding sites and as a result to have higher tumor uptake than agonists [24,25]. Exendin(9–39) isolated from *Heloderma suspectum* venom was identified as GLP-1 receptor antagonist binding with high affinity [26]. There are only few reports on radiolabeled Ex(9–39)-based tracers showing that Ex(9–39) derivatives radiolabeled with ^68^Ga [27] or ^18^F [28] are not suitable for imaging due to the low tumor uptake.

Our recent data also demonstrate that in contrast to Ex-4-based tracers, which tolerated various modifications at the C-terminal very well, the Ex(9–39) antagonist was very sensitive to conjugation of the ^68^Ga-DOTA or ^68^Ga-NODAGA complexes at the positions Lys^27^ or Lys^40^ [29]. The resulting probes had low affinity and were not suitable for GLP-1 R imaging [29]. However, Ex(9–39) radioiodinated with ^125^I at Lys^27^ using the Bolton Hunter (BH) method showed the same binding properties for the human GLP-1 receptor-expressing tissues *in vitro* as endogenous GLP-1 [30]. We demonstrated that *in vivo* ^125^I-BH-Ex(9–39) accumulated in the Ins-1E xenografts with an uptake similar to that of ^68^Ga-Ex-4, the GLP-1R tracer currently used in the clinic [22]. Most importantly, however radioiodination resulted in a significant reduction of the radioligand’s kidney uptake [29]. In comparison to ^68^Ga-Ex-4, the tumor-to-kidney ratio of ^125^I-BH-Ex-(9–39) improved 20-fold.

However, the Bolton-Hunter method is not so trivial to translate into the clinic. In the current study we selected the direct iodination approach, because it is a one-step process and the *in vivo* deiodination efficiency of ^125^I-Tyr is even higher than that for ^125^I-BH [31]. As a consequence the kidney uptake can be expected to be even lower for the directly iodinated peptides. Since radioiodination of Ex(9–39) with ^125^I-BH at position Lys^27^ was tolerated better than conjugation of ^68^Ga-DOTA or ^68^Ga-NODAGA in the same position, we hypothesized that radioiodination at the C-terminal Tyr^40^ may also potentially lead to a GLP-1R antagonist tracer with high affinity and favourable pharmacokinetics. In this study, the Ex-4 agonist and Ex(9–39) antagonist radioiodinated at the Tyr^40^ residue were evaluated *in vitro* and *in vivo* and compared side by side to the reference compound, [Nle^14^,Lys^40^(Ahx-DOTA-^68^Ga)NH_2_]Ex-4.

## Materials and Methods

### Reagents and instruments

All reagents and solvents were purchased from Sigma-Aldrich (Taufkirchen, Germany) and Carl Roth GmbH & Co. KG (Karlsruhe, Germany). The details for the analytical and semi-preparative HPLC set up are described in the S1 File.

### Peptides and radioiodination

[Nle^14^,Tyr^40^-NH_2_]Ex-4 and [Nle^14^,Tyr^40^-NH_2_]Ex-(9–39) precursor peptides were purchased from Biotrend Chemikalien GmbH (Köln, Germany). [Nle^14^,^125^I-Tyr^40^-NH_2_]Ex-4 and [Nle^14^,^125^I-Tyr^40^-NH_2_]Ex(9–39) were produced from precursor peptides by Celerion (Zürich, Switzerland) using the Chloramine-T method. [Nle^14^,^127^I-Tyr^40^-NH_2_]Ex-4 and [Nle^14^,^127^I-Tyr^40^-NH_2_]Ex(9–39), the corresponding non-radioactive reference peptides were custom-synthesized by Peptide Specialty Laboratories GmbH (Heidelberg, Germany) and Biotrend Chemikalien GmbH (Köln, Germany), respectively, using a Fmoc-protected 3-iodotyrosine as a building block.

[Nle^14^,^124^I-Tyr^40^-NH_2_]Ex-4 was prepared from [Nle^14^,Tyr^40^-NH_2_]Ex-4 using I-124 (Perkin-Elmer, Boston, USA) carrier-free (theoretical specific activity of I-124 is 1147 TBq/mmol), employing the Chloramine-T method following a published protocol [32] with minor modifications (for the details please see S2 File).

### *In vitro* binding assays

Ins-1E cells (a gift from Dr. Günter Päth) were maintained in RPMI medium, containing 10% FBS, 1mM sodium pyruvate, 50 μM ß-mercaptoethanol, 10 mM HEPES (pH 7.2), Penicillin G (100 IA/ml) and Streptomycin (10 mg/ml). One day prior to the experiments, cells (1.8 × 10^6^) were plated in triplicates in the 6-well plates. For the saturation binding experiment, cells were incubated with 3 kBq of [Nle^14^,^125^I-Tyr^40^-NH_2_]Ex-4 and increasing amounts of [Nle^14^, ^127^I-Tyr^40^-NH_2_]Ex-4 peptide (1–100 nM) in the growth medium, containing 1%FBS for 2 hours at +4°C. To determine the non-specific binding, three additional wells were blocked with 1 μM of [Nle^14^,^127^I-Tyr^40^-NH_2_]Ex-4. After washing with cold PBS cell bound activity was collected with 1M NaOH (3x1 ml). For internalization experiments, Ins-1E cells were incubated with 5 kBq of [Nle^14^,^125^I-Tyr^40^-NH_2_]Ex-4, containing 1 nM of [Nle^14^,^127^I-Tyr^40^-NH_2_]Ex-4 at 37°C. At specific time points the internalization was stopped by washing the cells with ice-cold PBS and the membrane bound and internalized activity were collected as described previously [33]. ^125^I-radioactivity in samples was measured using a gamma counter (Cobra 5003; Packard Instruments). The percentage of specifically bound activity per 1x10^6^ cells was assessed by comparison with standards and non-specific controls.

### Xenograft model and biodistribution studies

All animal experiments were conducted in accordance with the German animal protection law (TierSchG). The protocol was approved by the Animal Welfare Ethics committees of the University of Freiburg (Regierungspräsidium Freiburg Az G-12/21). Female Balb/c Nude mice (18–20 g, 6–8 weeks old) were obtained from Janvier Labs (Saint-Berthevin Cedex, France) and were housed and handled in accordance with the good animal practice as defined by FELASA and the national animal welfare body GVSOLAS. Xenografts were established on the right shoulder by s.c. injection of 7 million of Ins-1E cells in 1:1 v/v mixture of PBS and matrigel (final volume 100 μl). Mice were fed 60% Glucose Diet (PROVIMI KLIBA SA, Kaiseraugst, Switzerland).

Ins-1E tumor-bearing mice were administered with 40 kBq of [Nle^14^,^125^I-Tyr^40^-NH_2_]Ex-4 or [Nle^14^,^125^I-Tyr^40^-NH_2_]Ex(9–39) in 100 μL sterile saline *via* intravenous (*i*.*v*.) tail-vein injection. For mass dependence 10 or 100 pmol of [Nle^14^, ^127^I-Tyr^40^-NH_2_]Ex-4 or 10 pmol of [Nle^14^,^127^I-Tyr^40^-NH_2_]Ex(9–39) were coinjected together with the radioiodinated peptide. For the blocking experiment, 80 nmol of Ex(9–39) was injected 5 minutes before the administration of iodinated peptide. The sodium iodide symporters were blocked by Irenat (Bayer) (120 mg/kg, i.v.). Animals (*n* = 3–4, per group) were euthanized by asphyxiation with excess isoflurane at 1, 4 and 24 h post-radiotracer administration, and tissues were removed, rinsed in water, dried in air, weighed and counted on a calibrated and normalized gamma-counter.

### Small-animal PET/CT imaging

Mice were injected *iv* with [Nle^14^,^124^I-Tyr^40^-NH_2_]Ex-4 (2.5–3.0 MBq in 100 μL sterile saline). At 1, 2 and 4 hour post injection 20–40 minute static scans were acquired using microPET Focus 120 scanner (Concorde Microsystems), followed by 2 minute CT scans on micro-CT-Tomoscope Synergy (CT Imaging GmbH). PET sinograms were reconstructed using a 2-dimensional ordered-subset expectation maximization (2D-OSEM) algorithm. Image counts per pixel per second were calibrated to activity concentrations (Bq/mL) by measuring a 3.5 cm cylinder phantom filled with a known concentration of radioactivity. The PET and CT images were co-registered using the Rover software (ABX, Radeberg, Germany). PET images were analyzed using Rover software (ABX, Radeberg, Germany). The regions of interest were drawn based on the CT to include the entire tumor or the kidney and the mean %IA/g was calculated. For the time activity curves the PET images recorded at 1, 2 and 4 h p.i. were merged together and the mean %IA/g were calculated from the VOI placed in the same position in the tumor.

### Statistical analysis

A statistical analysis was performed using GraphPad Prism 5.01 (GraphPad Software, Inc) and Microsoft Excel. Data were analyzed by using the unpaired, two-tailed Student’s *t*-test.

## Results

### Peptides

The chemical purity of the precursor peptides for radioiodination, [Nle^14^,Tyr^40^-NH_2_]Ex-4 and [Nle^14^,Tyr^40^-NH_2_]Ex(9–39), and the non-radioactive reference peptides,[Nle^14^,^127^I-Tyr^40^-NH_2_]Ex-4 and [Nle^14^,^127^I-Tyr^40^-NH_2_]Ex(9–39) was determined by analytical HPLC to be >95%. Compounds were characterized by either ESI-MS or MALDI-MS and the obtained masses matched the exact calculated masses for all compounds (S1 Table).

### Radiochemical purity and *in vitro* characterization of [Nle^14^,^125^I-Tyr^40^-NH_2_]Ex-4 and [Nle^14^,^125^I-Tyr^40^-NH_2_]Ex(9–39)

[Nle^14^,^125^I-Tyr^40^-NH_2_]Ex-4 and [Nle^14^,^125^I-Tyr^40^-NH_2_]Ex(9–39) were delivered carrier-free, with a specific activity of 81.4 TBq/mmol and a radiochemical purity of > 95% (S1 and S2 Figs). The identity of the radioiodinated peptides was confirmed by coinjection with the corresponding non-radioactive reference peptides, containing ^127^I (S1 and S2 Figs).

**Fig 1:**
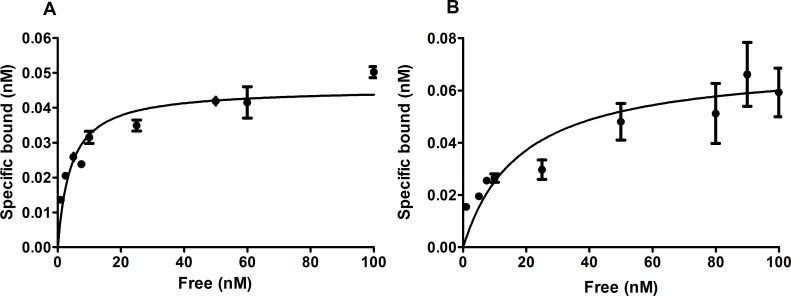
*In vitro* saturation binding data. The graphs show the percentage of specifically bound [Nle^14^,^125^I-Tyr^40^-NH_2_]Ex-4 (A) or [Nle^14^,^125^I-Tyr^40^-NH_2_]Ex(9–39) (B) per 1×10^6^ cells after incubation of Ins-1E cells with increasing amounts of the peptide. Values are mean±standard error of triplicate measurements.

**Fig 2:**
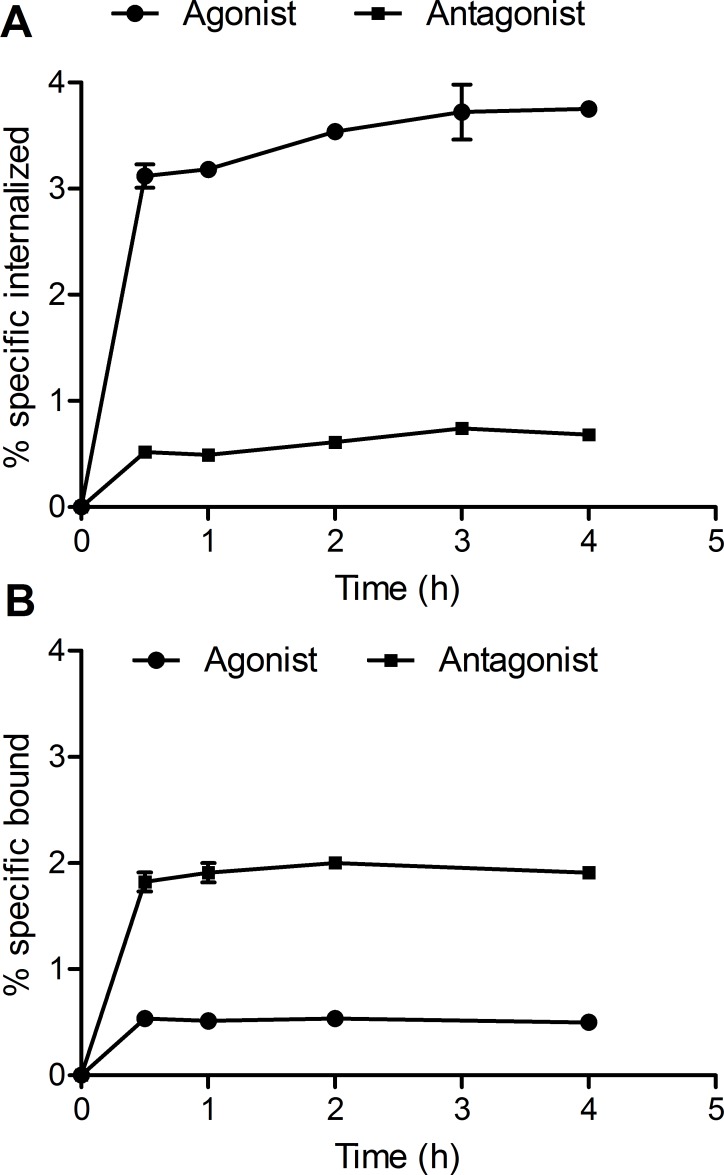
**Internalization (A) and binding (B) curves for [Nle**^**14**^**,**^**125**^**I-Tyr**^**40**^**-NH**_**2**_**]Ex-4 agonist and [Nle**^**14**^**,**^**125**^**I-Tyr**^**40**^**-NH**_**2**_**]Ex(9–39) antagonist in Ins-1E cells.** The plots show the percentage of specifically internalized and specifically bound peptide versus time. Values are mean±standard deviation of triplicate measurements.

The *in vitro* binding properties of the [Nle^14^,^125^I-Tyr^40^-NH_2_]Ex-4 agonist and [Nle^14^,^125^I-Tyr^40^-NH_2_]Ex(9–39) antagonist were studied using Ins-1E cells, expressing the rat GLP-1 receptor. The results of the saturation binding experiments demonstrate that [Nle^14^,^125^I-Tyr^40^-NH_2_]Ex-4 bound to Ins-1E cells with a K_d_ of 4.1±1.1 nM and a B_max_ of 0.045±0.002 nM (Fig 1A). [Nle^14^,^125^I-Tyr^40^-NH_2_]Ex(9–39), in contrast, had a K_d_ value of 18.1±6.9 nM and a B_max_ value of 0.071±0.007 nM (Fig 1B). Fig 2 shows cell binding and internalization kinetics of the [Nle^14^,^125^I-Tyr^40^-NH_2_]Ex-4 and [Nle^14^,^125^I-Tyr^40^-NH_2_]Ex(9–39) in Ins-1E cells. The amount of internalized [Nle^14^,^125^I-Tyr^40^-NH_2_]Ex-4 after 0.5 h at 37°C was 3.12±0.11% reaching 3.75±0.06% after 4 h (Fig 2A), while the amount of specifically bound [Nle^14^,^125^I-Tyr^40^-NH_2_]Ex-4 reached 0.53±0.01% after 0.5 h and remained the same until 4 hours (Fig 2B). The antagonist [Nle^14^,^125^I-Tyr^40^-NH_2_]Ex(9–39) showed very different kinetics with a significantly higher percentage of specifically bound peptide (1.82±0.09%) and a lower amount of internalized peptide (0.52±0.05%) at 0.5 h. After 4 h the amount of bound peptide remained the same (Fig 2B) and the amount of internalized peptide increased slightly to 0.68±0.03% (Fig 2A).

### Biodistribution of [Nle^14^,^125^I-Tyr^40^-NH_2_]Ex-4

At 1 hour post injection (p.i.) of [Nle^14^,^125^I-Tyr^40^-NH_2_]Ex-4 the highest uptake was found in the Ins-1E tumor (72.8±12.2%IA/g) followed by the lung (30.5±3.6%IA/g) and the pancreas (25.3±4.2%IA/g) (Table 1). Blocking with an excess of GLP-1 receptor antagonist reduced the uptake in the Ins-1E tumor, lung and pancreas by 92%, 93% and 93%, respectively. Additionally, 13.4±1.2%IA/g accumulated in the stomach and the blocking reduced the stomach uptake by 30%. Importantly, the kidney uptake was very low, only 7.5±0.7%IA/g, resulting in a very favourable tumor-to-kidney ratio of 9.7 at 1 hour p.i. (Table 1). The tumor-to-blood and tumor-to-muscle ratios at 1 hour p.i. were 25.1 and 182, respectively.

**Table 1:** Biodistribution of [Nle^14^,^125^I-Tyr^40^-NH_2_]Ex-4 in mice bearing Ins-1E xenografts.

Organ	%IA/g ^#^			
	1 h ^a^	1 h–blocked	4 h	24 h
**Blood**	2.9±0.2	2.9±0.5	2.2±0.2*	0.1±0.0**
**Heart**	1.3±0.2	0.7±0.1*	0.8±0.1*	0.1±0.0**
**Lung**	30.5±3.6	2.2±0.0**	8.8±1.8**	0.1±0.0**
**Liver**	1.5±0.1	1.1±0.3	0.8±0.0	0.1±0.0**
**Spleen**	1.5±0.3	1.1±0.2	1.1±0.1**	0.1±0.0**
**Pancreas**	25.3±4.2	1.9±0.1**	10.4±2.5**	0.9±0.5**
**Stomach**	13.4±1.2	9.3±0.6*	8.3±2.1*	0.3±0.0**
**Intestine**	3.9±0.5	1.3±0.2**	1.8±0.4**	0.1±0.0**
**Kidney**	7.5±0.7	5.7±0.1*	3.2±0.3**	0.2±0.0**
**Adrenal**	1.4±0.3	0.8±0.2	0.8±0.4	0.2±0.1**
**Muscle**	0.4±0.0	0.5±0.0	0.3±0.0*	0.1±0.0**
**Bone**	0.6±0.4	1.0±0.3	0.7±0.1	0.1±0.0
**Ins-1E**	72.8±12.2	5.9±0.6**	22.4±2.9**	3.7±2.5**
**Tumor-to-blood**	25.1		10.2	37
**Tumor-to-muscle**	182		74.7	37
**Tumor-to-kidney**	9.7		7.0	18.5
**Tumor-to-pancreas**	2.9		2.2	4.1

^**#**^ Values are mean±standard deviation (*n* = 3–4).

^a^-The peptide mass is 0.5 pmol, based on the specific activity of ^125^I.

*p<0.05.

**p<0.01, compared to 1h group.

After 4 hours p.i. there was a washout of activity from the target organs at a comparable rate with 22.4±2.9%IA/g, 10.4±2.5%IA/g, and 8.8±1.8%IA/g remaining in the tumor, pancreas and lung, respectively. Tumor-to-normal organ ratios at 4 hours p.i. were lower than at 1 hour p.i. (Table 1). At 24 hours p.i. most of the radioactivity was cleared from the body with 3.7±2.5%IA/g still remaining in the tumor (Table 1).

At one hour p.i. of [Nle^14^,^125^I-Tyr^40^-NH_2_]Ex-4 1.4±0.1%IA/organ was accumulated in the thyroid, confirming the dehalogenation of the tracer *in vivo*. However, blocking the sodium iodide symporter with irenat reduced the thyroid uptake by 94% (Table 2).

**Table 2:** Mass dependence of [Nle^14^,^125^I-Tyr^40^-NH_2_]Ex-4 biodistribution in nude mice bearing Ins-1E xenografts 1 h p.i.

Organ	%IA/g ^#^		
	10 pmol	%IA/g 100 pmol	10 pmol + irenat
**Blood**	3.9±0.2**	4.1±0.4**	4.1±0.4**
**Heart**	1.2±0.2	1.1±0.1	1.3±0.3
**Lung**	12.9±1.1**	5.5±0.8**	14.6±2.5**
**Liver**	1.4±0.1	1.5±0.1	1.6±0.2
**Spleen**	1.5±0.1	1.5±0.1	1.4±0.1
**Pancreas**	22.9±2.2	8.4±0.8**	19.9±5.9
**Stomach**	12.2±1.2	10.9±0.2*	5.3±0.9**
**Intestine**	2.6±0.7	1.7±0.1	2.1±0.2**
**Kidney**	7.9±1.1	7.5±0.8	8.5±1.9
**Adrenal**	1.2±0.5	0.9±0.2	1.2±0.1
**Muscle**	0.4±0.1	0.4±0.1	0.6±0.1
**Bone**	0.9±0.1	0.9±0.2	1.0±0.1
**Ins-1E**	56.3±8.1	20.3±3.5**	60.0±22.7
**Thyroid** ^**a**^	1.4±0.1^a^		0.1±0.0^a^**
**Tumor-to-blood**	14.4	5.0	14.6
**Tumor-to-muscle**	140.8	50.8	100.0
**Tumor-to-kidney**	7.1	2.7	7.1
**Tumor-to-pancreas**	2.5	2.4	3.0

^#^ Values are mean±standard deviation (*n* = 3–4).

*p<0.05.

**p<0.01, compared to the 1h / 0.5 pmol group of Table 1 (for the thyroid, comparison is against the 1h / 10pmol group of Table 2).

^a^ Thyroid uptake is reported as %IA/organ. Reason for this is the complicated delineation of the thyroid, which resulted in excision of variable amounts of adjacent tissue upon dissection and concomitantly incorrect values when uptake is reported as %IA/g.

Among the three studied peptide doses of [Nle^14^,^125^I-Tyr^40^-NH_2_]Ex-4 (0.5 pmol, 10 pmol and 100 pmol) the lowest peptide mass of 0.5 pmol corresponding to the highest specific activity showed the most favourable biodistribution profile with highest tumor uptake and highest tumor-to-normal organ ratios (Table 2).

### Biodistribution of [Nle^14^,^125^I-Tyr^40^-NH_2_]Ex(9–39)

Corresponding to the lower affinity of the antagonist, also the tumor uptake of [Nle^14^,^125^I-Tyr^40^-NH_2_]Ex(9–39) was lower, 12.7±4.1%IA/g at 1 h p.i. and it decreased after 4 h to 1.9±0.5%IA/g, similar to the activity in the blood pool (Table 3). The uptake in other receptor-expressing organs was also lower (Table 3). Blocking with an excess of cold antagonist reduced the uptake in the tumor, pancreas and lung by 80%, 93% and 88% respectively (Table 3). The uptake in the kidney at 1 h p.i. was also very low (7.6±1.2%IA/g) and identical to the agonist kidney uptake. However, due to the lower tumor uptake, the tumor-to-kidney ratio dropped to 1.7. The tumor-to-blood and tumor-to muscle ratios of [Nle^14^,^125^I-Tyr^40^-NH_2_]Ex(9–39) were substantially lower than the ratios for the agonist. The uptake in the thyroid was 1.3±0.2%IA/organ at 1 h p.i., indicating tracer deiodination *in vivo*. In terms of the peptide dose, the lowest dose of 0.5 pmol yielded the highest tumor uptake and best tumor-to-normal organ ratios (Table 3).

**Table 3:** Biodistribution of [Nle^14^,^125^I-Tyr^40^-NH_2_]Ex(9–39) in mice bearing Ins-1E xenografts.

Organ	%IA/g ^#^			
	1 h^a^	1 h–blocked	1 h– 10 pmol	4 h
**Blood**	4.2±0.5	3.2±0.2	4.4±0.1	2.0±0.4*
**Heart**	1.3±0.2	0.9±01	1.2±0.0	0.5±0.1*
**Lung**	13.6±1.0	1.7±0.4**	9.8±0.9*	3.3±1.2**
**Liver**	3.2±0.5	1.5±0.7*	2.9±0.3	0.9±0.2**
**Spleen**	1.5±0.2	1.1±0.1	1.7±0.1	0.8±0.1*
**Pancreas**	17.93±1.41	1.3±0.4**	17.8±2.4	3.6±1.2**
**Stomach**	11.9±1.6	12.0±0.7	11.2±3.6	5.3±1.9*
**Intestine**	2.4±0.4	2.2±0.5	3.8±1.9	1.1±0.0*
**Kidney**	7.6±1.2	4.2±0.2*	7.2±0.6	2.0±0.3**
**Adrenal**	1.3±0.4	0.6±0.5	1.3±0.1	0.5±0.1*
**Muscle**	0.7±0.2	0.6±0.0	1.2±0.7	0.6±0.4
**Bone**	1.2±0.1	1.4±0.6	1.3±0.2	0.8±0.3*
**Ins-1E**	12.7±4.1	2.6±0.8*	9.5±2.7	1.9±0.5*
**Thyroid**^**b**^	n.d	n.d.	1.3±0.2	n.d.
**Tumor-to-Blood**	3.1		2.2	1.0
**Tumor-to-Muscle**	17.8		7.7	3.3
**Tumor-to-Kidney**	1.7		1.3	1.0
**Tumor-to-pancreas**	0.7		0.5	0.5

^#^ Values are mean±standard deviation (*n* = 3–4).

^a^ the peptide mass is 0.5 pmol, based on the specific activity of ^125^I^.^

^b^—thyroid uptake is reported as %IA/organ.

*p<0.05.

**p<0.001, compared to the 1 h group.

n.d.—not determined.

### Biodistribution of the [Nle^14^,Lys^40^(Ahx-DOTA-^68^Ga)NH_2_]Ex-4 reference tracer

For the direct comparison, we studied the biodistribution of the ^68^Ga-labelled Ex-4 tracer in the same tumor model. At 1 h p.i. of 10 pmol of [Nle^14^,Lys^40^(Ahx-DOTA-^68^Ga)NH_2_]Ex-4, the kidney had the highest accumulation of activity (201.3±30.6%IA/g) followed by the Ins-1E tumor (58.3±15.6% IA/g), the pancreas (25.5±5.2% IA/g), and the lung (14.3±1.2%IA/g). The tumor-to-blood and tumor-to-muscle ratios were 48.5 and 215.9, respectively (Table 4). The tumor-to-kidney ratio was very low, only 0.3.

**Table 4:** Biodistribution of 10 pmol of [Nle^14^,Tyr^40^(Ahx-DOTA-^68^Ga)NH_2_] in mice bearing Ins-1E xenografts at 1 h p.i.

Organ	%IA/g ^#^
**Blood**	1.2±0.3
**Heart**	0.7±0.1
**Lung**	14.3±1.2
**Liver**	2.1±0.3
**Spleen**	1.9±0.7
**Pancreas**	25.5±5.2
**Stomach**	5.8±1.8
**Intestine**	1.9±0.4
**Kidney**	201.3±30.6
**Adrenal**	2.2±0.9
**Muscle**	0.3±0.2
**Bone**	0.610.2
**Ins-1E**	58.3±15.6
**Tumor-to-Blood**	48.6
**Tumor-to-Muscle**	215.9
**Tumor-to-Kidney**	0.3
**Tumor-to-Pancreas**	2.3

^#^ Values are mean±standard deviation (*n* = 3–4).

### Synthesis and *in vitro* characterization of [Nle^14^,^124^I-Tyr^40^-NH_2_]Ex-4

**Fig 3:**
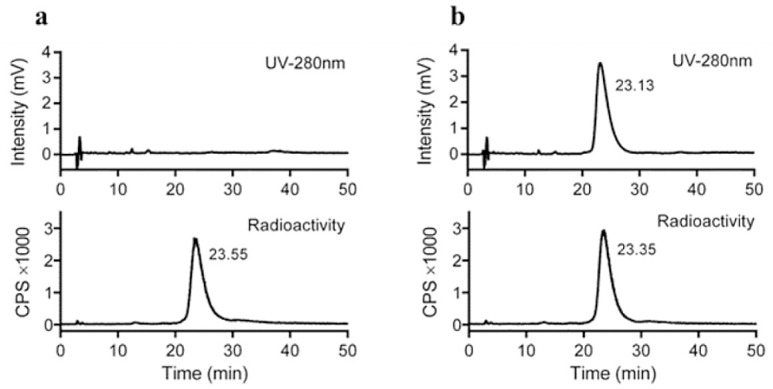
**Radio-HPLC showing the radiochemical purity of formulated [Nle**^**14**^**,**^**124**^**I-Tyr**^**40**^**-NH**_**2**_**]Ex-4 without (A) and with co-injection of cold reference [Nle**^**14**^**,**^**127**^**I-Tyr**^**40**^**-NH**_**2**_**]Ex-4 (B)**. UV-and radio-detectors were in series, resulting in a lag time of about 15 sec for the radiotrace. Numbers in the chromatograms refer to the peak retention time in minutes.

Given the high tumor uptake and excellent tumor-to-kidney ratios obtained with [Nle^14^,^125^I-Tyr^40^-NH_2_]Ex-4, we proceeded to prepare the corresponding ^124^I-labeled analogue, which would be suitable for PET-imaging studies. Radiosynthesis was performed using the chloramine-T method. The semi-preparative HPLC chromatogram of the crude [Nle^14^,^124^I-Tyr^40^-NH_2_]Ex-4 (S3 Fig) demonstrates that the peptide precursor [Nle^14^,Tyr^40^-NH_2_]Ex-4 elutes about 3 min earlier than the iodinated product [Nle^14^,^124^I-Tyr^40^-NH_2_]Ex-4, enabling isolation of the pure radio-iodinated tracer with high specific activity (S3 Fig). The collected product fractions from several syntheses were combined, trapped on a C8-SepPak, and eluted with ethanol to yield formulated [Nle^14^,^124^I-Tyr^40^-NH_2_]Ex-4 in 18.0±1.6% (*n* = 3) radiochemical yield and >95% radiochemical purity (Fig 3A and 3B).

**Fig 4:**
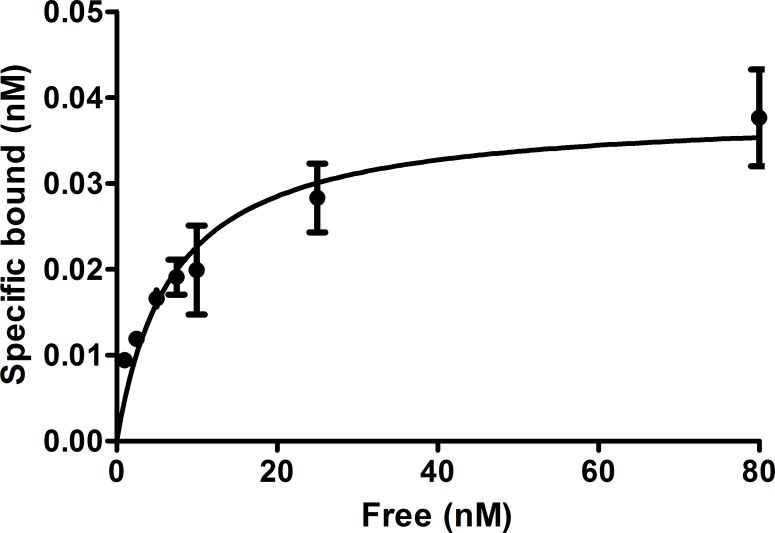
*In vitro* saturation binding data. **The curves show the percent of specifically bound [Nle**^**14**^**,**^**124**^**I-Tyr**^**40**^**-NH**_**2**_**]Ex-4 after incubation of Ins-1E cells with increasing amounts of peptide.** Values are mean±standard deviation of 3 replicates.

*In vitro* evaluation revealed that [Nle^14^,^124^I-Tyr^40^-NH_2_]Ex-4 bound to the cells with a K_d_ of 6.5±1.7 nM and a B_max_ of 0.038±0.003 nM (Fig 4).

### PET imaging of GLP-1 receptors in mice using [Nle^14^,^124^I-Tyr^40^-NH_2_]Ex-4

**Fig 5:**
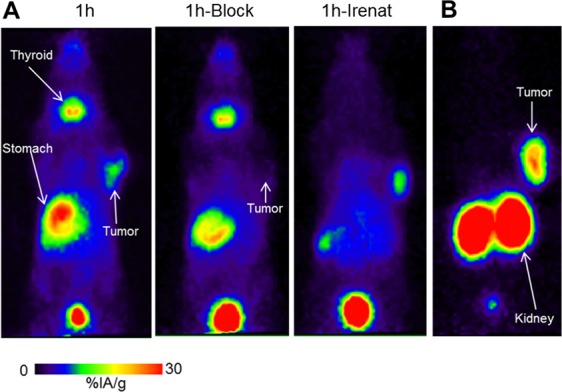
PET images of [Nle^14^,^124^I-Tyr^40^-NH_2_]Ex-4 and [Nle^14^,Lys^40^(Ahx-DOTA-^68^Ga)NH_2_]Ex-4 distribution in Ins-1E tumor-bearing mice. Image data are presented as maximum intensity projections (MIP) at 1 h post injection of [Nle^14^,^124^I-Tyr^40^-NH_2_]Ex-4 (**A**) or Nle^14^,Lys^40^(Ahx-DOTA-^68^Ga)NH_2_]Ex-4 (**B**). The middle panel shows an image after blocking the non-specific uptake; the right panel shows an image after blocking the sodium iodide symporter with irenat.

The *in vivo* properties of [Nle^14^,^124^I-Tyr^40^-NH_2_]Ex-4 were evaluated using PET imaging. Maximum-Intensity-Projection (MIP) PET images (Fig 5A, 1^st^ panel) demonstrate that at 1 hour p.i. of [Nle^14^,^124^I-Tyr^40^-NH_2_]Ex-4, the highest specific uptake was detected in Ins-1E tumor (10.8%IA/g based on image quantification). In the blocked mouse (Fig 5A, 2^nd^ panel) the tumor uptake was inhibited by 82% (1.9%IA/g left in the tumor). Apart from the bladder, high accumulation of radioactivity was also found in the thyroid (19.7%IA/g) and the stomach (28.3%IA/g) confirming the tracer radiodeiodination *in vivo*. The stomach uptake was partially inhibited in the blocked mouse, suggesting some GLP-1 receptor mediated uptake in the stomach, which was also described by others in the field. Blocking of the sodium iodide symporter with irenat did not affect the tumor uptake (11.4%IA/g) but completely inhibited the thyroid uptake and most of the stomach uptake (Fig 5A, 3^rd^ panel).

The high amount of radioactivity in the bladder already after 1 hour p.i. suggests that the tracer or its radiometabolites are cleared through the kidney. Finally, HPLC analysis of the urine taken at 1 and 4 h p.i. of [Nle^14^,^124^I-Tyr^40^-NH_2_]Ex-4 revealed that there was only free ^124^I-iodide and no intact peptide present in the mouse urine (S4 Fig).

**Fig 6:**
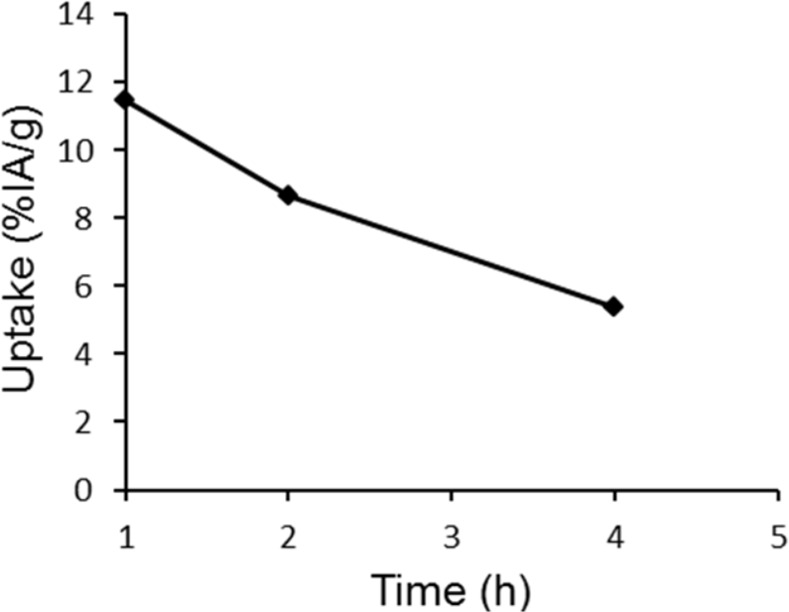
[Nle^14^,^124^I-Tyr^40^-NH_2_]Ex-4 tumor time-activity curves. The curves are derived from the PET image analysis and show the tumor uptake of [Nle^14^,^124^I-Tyr^40^-NH_2_]Ex-4 versus time.

The tumor time-activity curve derived from quantification of the PET images recorded at 1, 2, and 4 hours p.i. of irenat and [(Nle^14^,^124^I-Tyr^40^)NH_2_]Ex-4 shows a decrease in the tumor radioactivity from 11.4%IA/g at 1 h p.i. to 5.4%IA/g at 4h p.i. (Fig 6).

The MIP PET image of the reference GLP-1 receptor imaging agent [Nle^14^,Lys^40^(Ahx-DOTA-^68^Ga)NH_2_]Ex-4 at 1 h p.i (Fig 5B) demonstrates a typical biodistribution profile for the radiometal labelled Ex-4 analogue with a high tumor uptake but also very high uptake in the kidney.

## Discussion

GLP-1 receptor targeting peptides are important imaging tools for preoperative localization of benign insulinoma. Currently available tracers show a high and persistent renal uptake and low specific activity, resulting in side effects such as hypoglycaemia due to insulin secretion after activation of the GLP-1 receptor. We attempted to solve these issues by using radioiodinated analogues of the agonist Ex-4 and the antagonist Ex(9–39).

[Nle^14^,^125^I-Tyr^40^-NH_2_]Ex-4 showed high affinity binding to Ins-1E cells *in vitro* with a K_d_ of 4.1±1.1 nM. The K_d_ value for [Nle^14^,^125^I-Tyr^40^-NH_2_]Ex(9–39), however was 4.4 fold higher, demonstrating a significantly lower affinity towards GLP-1R. In contrast to the radiometal-labelled GLP-1R tracers, the fraction of internalized [Nle^14^,^125^I-Tyr^40^-NH_2_]Ex-4 did not increase with time after reaching the maximum of 3% at 0.5 h. This phenomenon has been previously described for radioiodinated tracers [9] and is related to the intracellular degradation and release of the main catabolite ^125^I-Tyr, which is not trapped in the lysosomal compartment as do the radiometal-containing catabolites [34]. As a result, some steady state between the amount of internalized radioactivity and the amount of released radioactivity is reached.

[Nle^14^,^125^I-Tyr^40^-NH_2_]Ex(9–39) behaved in *in vitro* binding assays as expected from an antagonist. In comparison to the agonist [Nle^14^,^125^I-Tyr^40^-NH_2_]Ex-4, it showed higher cell membrane binding but a 5.5-fold-lower internalization into Ins-1E cells. Interestingly, the Bmax value for the iodinated antagonist was higher (0.070±0.007 versus 0.045±0.003, p<0.05), potentially indicating that it recognizes more binding sites than the iodinated agonist. This feature of the antagonist tracer has been described for the SST-2 receptors, where antagonists recognized 10–15 fold more binding sites, compared to the agonists [24]. However, in spite of the higher number of binding sites, the lower affinity and lower internalization resulted in a lower total cellular accumulation of the GLP-1R antagonist (2.53%) in comparison to the GLP-1R agonist (4.43%).

*In vivo*, [Nle^14^,^125^I-Tyr^40^-NH_2_]Ex-4 demonstrated high and specific tumor uptake, which was equal to the tumor uptake of ^68^Ga-Ex-4. But most importantly the kidney uptake was very low resulting in the high tumor-to-kidney ratio of 9.7. This ratio was even better than the ratio of 3.5, which we reported for the antagonist ^125^I-BH-Ex(9–39) [29], and was the highest among the published GLP-1R tracers. The tumor-to-pancreas ratios were also very similar for [Nle^14^,^125^I-Tyr^40^-NH_2_]Ex-4 and ^68^Ga-Ex-4, which would translate into similar imaging contrast for insulinomas against the normal pancreas.

Somewhat surprisingly, the tumor uptake of the antagonist [Nle^14^,^125^I-Tyr^40-^NH_2_]Ex(9–39) was significantly lower, only 12.7±4.05%IA/g at 1 h p.i. and the activity was completely washed out after 4 h p.i. This may be explained by the higher K_d_ value of the compound. The kidney uptake of the iodinated antagonist was identical to the agonist (7.63±1.21%IA/g and 7.51±0.73%IA/g, respectively), but the tumor-to-kidney ratio and other tumor-to-normal organ ratios were much lower.

The mechanism behind the low kidney retention of ^125^I-Ex-4 is related to the non-residualizing nature of the ^125^I label [34]. After reabsorption and lysosomal degradation in the proximal tubular cells of the kidneys, the main catabolite ^125^I-Tyr, is not retained in the lysosomes but instead freely diffuses out of the cells, and the iodide released upon deiodination rapidly accumulates in the thyroid tissue and stomach via the sodium iodide symporter. Indeed, the radioactivity in the thyroid and stomach for both tracers, was effectively blocked by inhibiting the sodium iodide symporter with irenat.

As we hypothesized, the kidney uptake of the directly iodinated [Nle^14^,^125^I-Tyr^40^-NH_2_]Ex-4 and [Nle^14^,^125^I-Tyr^40^-NH_2_]Ex(9–39) (7.5%IA/g) at 1 h p.i. was even lower than that of the Bolton-Hunter-labelled analogue ^125^I-BH-Ex(9–39) (12.1±1.4%IA/g) [29]. Higher accumulation of radioactivity in the stomach and the thyroid after injection of [Nle^14^,^125^I-Tyr^40^-NH_2_]Ex-4 and [Nle^14^,^125^I-Tyr^40^-NH_2_]Ex(9–39) in comparison to ^125^I-BH-Ex(9–39) [29] confirms more efficient dehalogenation for the directly iodinated peptides. The relatively fast washout of activity from the tumor within 4 hours for the directly iodinated agonist can also be explained by *in vivo* dehalogenation. Since fast washout of activity from normal organs will result in a lower radiation exposure, the ^124^I congener of the directly iodinated Ex-4 agonist may prove a valuable candidate for clinical PET imaging, provided that an early time-point is chosen for imaging. However, relatively fast reduction in the tumor uptake of iodinated Ex-4 would limit the time window for intraoperative localization of insulinoma using a surgical probe, compared to the In-111-labeled Ex-4 analogs used in clinic. Based on the tumor uptake and tumor-to-normal organ ratios, the optimal imaging time point for the iodinated Ex-4 tracer would be at 1 hour p.i., which is similar to ^68^Ga-Ex-4.

As a proof-of-concept, the ^124^I analogue of the most promising tracer candidate, the agonist [Nle^14^,^124^I-Tyr^40^-NH_2_]Ex-4, was evaluated in PET-studies. Although absolute tumor uptake levels were somewhat lower than expected, the PET-images confirmed the very favourable biodistribution profile of the ^124^I congener in comparison to ^68^Ga-Ex-4, with a pronounced and specific tumor uptake and low kidney retention. We strongly believe in the potential of ^124^I- and ^123^I-labeled Ex-4 derivatives for GLP-1 targeted imaging and currently develop automated labelling strategies. The goal is production of these tracers in high radiochemical purity, specific activity and improved radiochemical yields, which is an important factor given the high cost of ^123^I and ^124^I in particular, and the activities of these tracers needed for comprehensive imaging studies in animals and finally for clinical translation.

## Conclusions

Even though the antagonist [Nle^14^,^125^I-Tyr^40^NH_2_]Ex(9–39) recognized more binding sites on GLP-1R, it had a lower affinity than the agonist and it may not be suitable for clinical translation. The [Nle^14^,^125^I-Tyr^40^NH_2_]Ex-4 agonist, in contrast, showed excellent tumor uptake and at the same time exhibited pharmacokinetics superior to all other GLP-1R tracers presently available, with particularly high tumor-to-kidney ratio and good contrast to normal organs. Preclinical PET imaging data strongly suggest that Ex-4 radioiodinated with ^124^I may be a promising alternative to the radio-metal labelled derivatives for imaging of GLP-1 receptor positive insulinoma. It can further improve the sensitivity of the preoperative localization of benign insulinoma.

## Supporting Information

S1 Fig **Analytical HPLC chromatogram of [Nle**^**14**^**,**^**125**^**I-Tyr**^**40**^**-NH**_**2**_**]Ex-4 without (A) and with co-injection of cold reference [Nle**^**14**^**,**^**127**^**I-Tyr**^**40**^**-NH**_**2**_**]Ex-4 (B).** UV-and radio-detectors were in series, resulting in a lag time of about 15 sec for the radiotrace. Numbers in the chromatograms refer to peak retention time in minutes.
(PDF)

S2 Fig **Analytical HPLC chromatogram of [Nle**^**14**^**,**^**125**^**I-Tyr**^**40**^**NH**_**2**_**]Ex-(9–39) without (A) and with co-injection of cold reference [Nle**^**14**^**,**^**127**^**I-Tyr**^**40**^**-NH**_**2**_**]Ex(9–39) (B).** UV-and radio-detectors were in series, resulting in a lag time of about 15 sec for the radiotrace. Numbers in the chromatograms refer to peak retention time in minutes. The peak at 13.88 min originates from bovine serum albumin (BSA) employed for formulation of the radiotracer.
(PDF)

S3 Fig Typical semi-preparative HPLC chromatogram of crude [Nle^14^,^124^I-Tyr^40^-NH_2_]Ex-4. UV-and radio-detectors were in series, with the radio-detector preceding the UV-detector. Numbers in the chromatograms refer to peak retention time in minutes. The product peak was collected in several fractions (based on the radio-trace and before entering the UV-detector), and fractions containing the pure product (as determined by analytical HPLC analysis of individual fractions) were pooled, trapped on a C8-SepPak and formulated in EtOH. Note that the peptide precursor [Nle^14^,Tyr^40^-NH_2_]Ex-4 elutes about 3 min earlier than the iodinated product [Nle^14^,^124^I-Tyr^40^-NH_2_]Ex-4, enabling isolation of the pure radio-iodinated tracer in high specific activity.
(PDF)

S4 Fig **Analytical HPLC chromatograms from the urine of mice obtained at 1h (A) and 4h (B) post *iv* injection of [Nle**^**14**^**,**^**124**^**I-Tyr**^**40**^**-NH**_**2**_**]Ex-4.** Note that apart from ^124^I-iodide (peak at 2.38 min), neither the intact tracer (HPLC retention time ~23 min; see Fig 3 in the main manuscript) nor other radioactive metabolites were found.
(PDF)

S1 File Analytical and semi-preparative HPLC. (PDF)

S2 File Preparation of [Nle^14^,^124^I-Tyr^40^-NH_2_]Ex-4. (PDF)

S1 Table Mass spectrometry data of peptides used in this study. (PDF)
