# Supplementary material for: Radioiodinated Exendin-4 Is Superior to the Radiometal-Labelled Glucagon-Like Peptide-1 Receptor Probes Overcoming Their High Kidney Uptake
Source: PLoS One. 2017 Jan 19;12(1):e0170435. doi: 10.1371/journal.pone.0170435 (PMC5245897; doi:10.1371/journal.pone.0170435)

S1 Fig. Analytical HPLC chromatogram of [Nle^{14},^{125}I-Tyr^{40}-NH_2]Ex-4 without (A) and with co-injection of cold reference [Nle^{14},^{127}I-Tyr^{40}-NH_2]Ex-4 (B). UV-and radio-detectors were in series, resulting in a lag time of about 15 sec for the radiotrace. Numbers in the chromatograms refer to peak retention time in minutes.

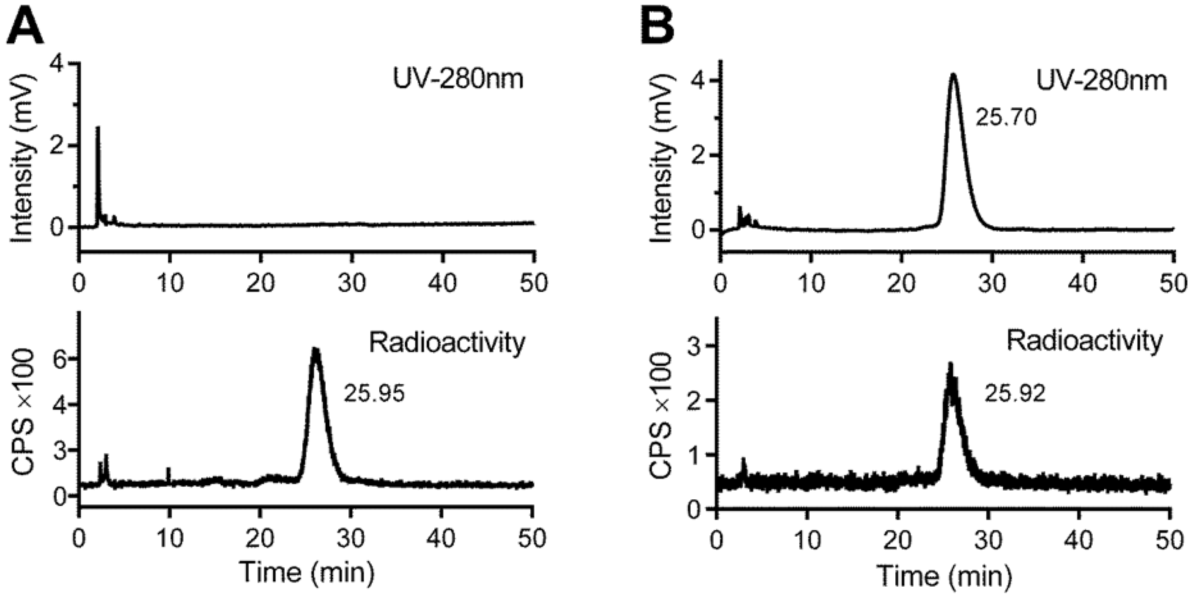

Supplement: S1 Fig — Analytical HPLC chromatogram of [Nle14,125I-Tyr40-NH2]Ex-4 without (A) and with co-injection of cold reference [Nle14,127I-Tyr40-NH2]Ex-4 (B). UV-and radio-detectors were in series, resulting in a lag time of about 15 sec for the radiotrace. Numbers in the chromatograms refer to peak retention time in minutes. (PDF) [file pone.0170435.s001.pdf]
