# Supplementary material for: Radioiodinated Exendin-4 Is Superior to the Radiometal-Labelled Glucagon-Like Peptide-1 Receptor Probes Overcoming Their High Kidney Uptake
Source: PLoS One. 2017 Jan 19;12(1):e0170435. doi: 10.1371/journal.pone.0170435 (PMC5245897; doi:10.1371/journal.pone.0170435)

S2 Fig. Analytical HPLC chromatogram of [Nle^{14},^{125}I-$Tyr^{40}$$NH_2$]Ex-(9-39) without (A) and with co-injection of cold reference [Nle^{14},^{127}I-Tyr^{40}-NH_2]Ex(9-39) (B). UV-and radio-detectors were in series, resulting in a lag time of about 15 sec for the radiotrace. Numbers in the chromatograms refer to peak retention time in minutes. The peak at 13.88 min originates from bovine serum albumin (BSA) employed for formulation of the radiotracer.

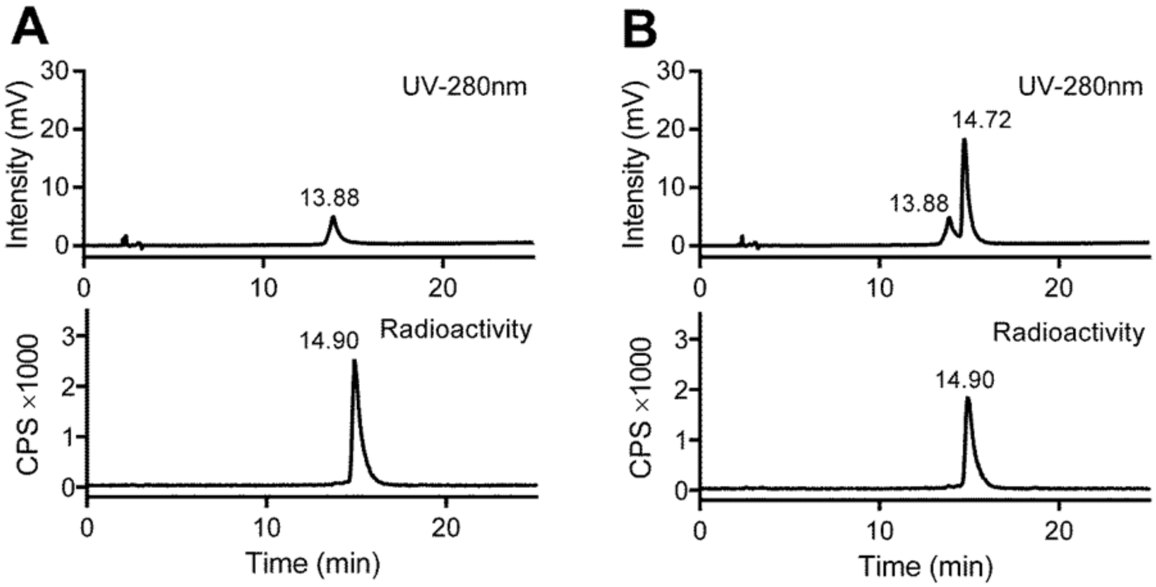

Supplement: S2 Fig — Analytical HPLC chromatogram of [Nle14,125I-Tyr40NH2]Ex-(9–39) without (A) and with co-injection of cold reference [Nle14,127I-Tyr40-NH2]Ex(9–39) (B). UV-and radio-detectors were in series, resulting in a lag time of about 15 sec for the radiotrace. Numbers in the chromatograms refer to peak retention time in minutes. The peak at 13.88 min originates from bovine serum albumin (BSA) employed for formulation of the radiotracer. (PDF) [file pone.0170435.s002.pdf]
