# Supplementary material for: Radioiodinated Exendin-4 Is Superior to the Radiometal-Labelled Glucagon-Like Peptide-1 Receptor Probes Overcoming Their High Kidney Uptake
Source: PLoS One. 2017 Jan 19;12(1):e0170435. doi: 10.1371/journal.pone.0170435 (PMC5245897; doi:10.1371/journal.pone.0170435)

S3 Fig. Typical semi-preparative HPLC chromatogram of crude [Nle^{14},^{124}I-Tyr^{40}-NH_2]Ex-4. UV-and radio-detectors were in series, with the radio-detector preceding the UV-detector. Numbers in the chromatograms refer to peak retention time in minutes. The product peak was collected in several fractions (based on the radio-trace and before entering the UV-detector), and fractions containing the pure product (as determined by analytical HPLC analysis of individual fractions) were pooled, trapped on a C8-SepPak and formulated in EtOH. Note that the peptide precursor [Nle^{14},Tyr^{40}-NH_2]Ex-4 elutes about 3 min earlier than the iodinated product [Nle^{14},^{124}I-Tyr^{40}-NH_2]Ex-4, enabling isolation of the pure radio-iodinated tracer in high specific activity.

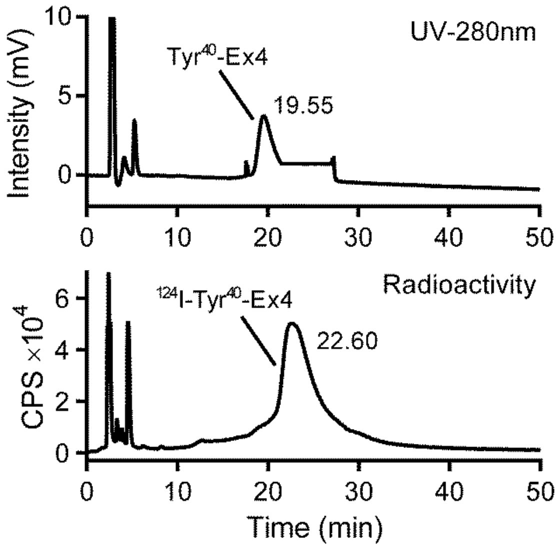

Supplement: S3 Fig — UV-and radio-detectors were in series, with the radio-detector preceding the UV-detector. Numbers in the chromatograms refer to peak retention time in minutes. The product peak was collected in several fractions (based on the radio-trace and before entering the UV-detector), and fractions containing the pure product (as determined by analytical HPLC analysis of individual fractions) were pooled, trapped on a C8-SepPak and formulated in EtOH. Note that the peptide precursor [Nle14,Tyr40-NH2]Ex-4 elutes about 3 min earlier than the iodinated product [Nle14,124I-Tyr40-NH2]Ex-4, enabling isolation of the pure radio-iodinated tracer in high specific activity. (PDF) [file pone.0170435.s003.pdf]
