# Supplementary material for: Radioiodinated Exendin-4 Is Superior to the Radiometal-Labelled Glucagon-Like Peptide-1 Receptor Probes Overcoming Their High Kidney Uptake
Source: PLoS One. 2017 Jan 19;12(1):e0170435. doi: 10.1371/journal.pone.0170435 (PMC5245897; doi:10.1371/journal.pone.0170435)

S4 Fig. Analytical HPLC chromatograms from the urine of mice obtained at 1h (A) and 4h (B) post *iv* injection of [Nle^{14},^{124}I-Tyr^{40}-NH_2]Ex-4. Note that apart from ^{124}I-iodide (peak at 2.38 min), neither the intact tracer (HPLC retention time ~23 min; see Fig. 3 in the main manuscript) nor other radioactive metabolites were found.

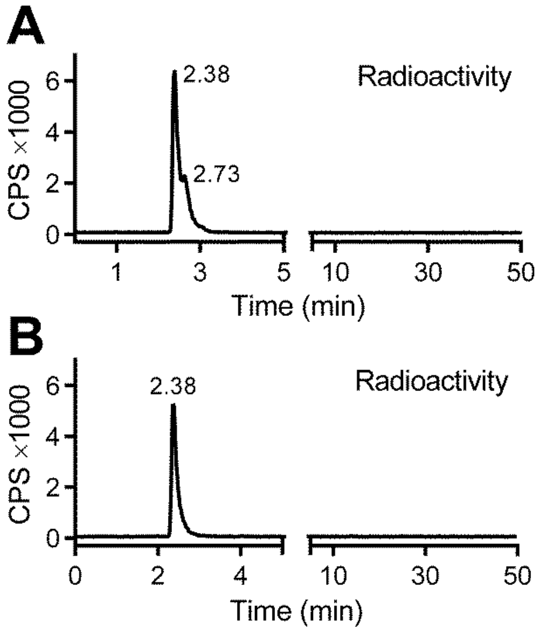

Supplement: S4 Fig — Analytical HPLC chromatograms from the urine of mice obtained at 1h (A) and 4h (B) post iv injection of [Nle14,124I-Tyr40-NH2]Ex-4. Note that apart from 124I-iodide (peak at 2.38 min), neither the intact tracer (HPLC retention time ~23 min; see Fig 3 in the main manuscript) nor other radioactive metabolites were found. (PDF) [file pone.0170435.s004.pdf]
