# Supplementary material for: Radioiodinated Exendin-4 Is Superior to the Radiometal-Labelled Glucagon-Like Peptide-1 Receptor Probes Overcoming Their High Kidney Uptake
Source: PLoS One. 2017 Jan 19;12(1):e0170435. doi: 10.1371/journal.pone.0170435 (PMC5245897; doi:10.1371/journal.pone.0170435)
Supplement: S1 File — (PDF) [file pone.0170435.s005.pdf]

S 1 File. Analytical and semi-preparative HPLC.

Semi-preparative HPLC for purification of the nonradioactive peptides was performed on a Knauer Smartline 1000 HPLC system equipped with a VP250/21 Nucleosil 120-5 C18 column from Macherey-Nagel (Düren, Germany), mobile phase 42% acetonitrile/water (0.1% TFA), flow 12 mL/min, UV-detection at 280 nm. Quality control of [Nle^{14},$^{124/125}I$-Tyr^{40}-NH_2]Ex-4 as well as the corresponding non-radioactive precursor and reference peptides was performed by analytical RP-HPLC on an identical system additionally equipped with a Ramona Star radioactivity detector (Raytest GmbH, Straubenhardt, Germany) using an EC250/4.6 Nucleosil 100-7 C2 column from Macherey-Nagel (Düren, Germany) with 33.5% acetonitrile/water (0.1% TFA) as mobile phase, flow 1.2 mL/min, UV-detection at 280 nm. Quality control of [Nle^{14},$^{124/125}I$-Tyr^{40}-NH_2]Ex(9-39) as well as the corresponding non-radioactive precursor and reference peptides was performed by analytical RP-HPLC on an Agilent 1260 Infinity system equipped with an Agilent 1200 DAD UV-detector (UV detection at 280 nm) and a Ramona Star radioactivity detector (Raytest GmbH, Straubenhardt, Germany) using an EC250/4.6 Nucleosil 100-5 C18 column from Macherey-Nagel (Düren, Germany) and a gradient of 30-60% acetonitrile/water (0.1% TFA) in 25 min with a flow of 1.0 mL/min and UV-detection at 280 nm. Mass spectra were acquired on a Thermo Scientific Exactive mass spectrometer (ESI) and a Bruker Daltonics Ultraflex TOF/TOF (MALDI).
