# Supplementary material for: Radioiodinated Exendin-4 Is Superior to the Radiometal-Labelled Glucagon-Like Peptide-1 Receptor Probes Overcoming Their High Kidney Uptake
Source: PLoS One. 2017 Jan 19;12(1):e0170435. doi: 10.1371/journal.pone.0170435 (PMC5245897; doi:10.1371/journal.pone.0170435)
Supplement: S2 File — (PDF) [file pone.0170435.s006.pdf]

S 2 File. Preparation of [Nle^{14},^{124}I-Tyr^{40}-NH_2]Ex-4

The ^{124}I-iodide in 0.02 M NaOH (35 – 44 µL, 43 – 52 MBq) and a 0.54 µg/µL solution of chloramine-T in sodium phosphate buffer pH 7.4 (20 µL) were sequentially added to a mixture of 0.5 M sodium phosphate buffer pH 7.4 (20 µL) and a 1 µg/µL solution of [Nle^{14},Tyr^{40}-NH_2]Ex-4 in water (8.7 µL, 2 µmol) . After short mixing with the pipet and incubation at room temperature for 2 minutes, the reaction was quenched by addition of 4.8 µg/µL aqueous $Na_2S_2O_5$ (6 µL), and directly subjected to semi-preparative HPLC. Column: EC250/4.6 Nucleosil 100-7 C2, Macherey-Nagel (Düren, Germany), mobile phase 33.5% acetonitrile/water (0.1% TFA), flow 1.2 mL/min, UV-detection at 280 nm. Fractions (1 min/fraction) were collected after 20 min when the main peak started to elute. Fractions containing the pure product were pooled (pure product typically eluted between 22 min and 25 min; individual fractions were analyzed on analytical HPLC before combining as required), 10-fold diluted with water, and passed over a Sep-Pak® C_8 Plus Light cartridge (Waters GmbH, Eschborn, Germany), which had been preconditioned with ethanol and water. After rinsing with water (10 mL), the purified radiotracer was eluted with absolute ethanol (0.7 mL) into an Eppendorf tube and evaporated at 40°C under a stream of nitrogen to a final volume of 30 – 60 µL, which was then further diluted as required for the *in vitro* and *in vivo* studies.
