# Supplementary material for: Radioiodinated Exendin-4 Is Superior to the Radiometal-Labelled Glucagon-Like Peptide-1 Receptor Probes Overcoming Their High Kidney Uptake
Source: PLoS One. 2017 Jan 19;12(1):e0170435. doi: 10.1371/journal.pone.0170435 (PMC5245897; doi:10.1371/journal.pone.0170435)
Supplement: S1 Table — (PDF) [file pone.0170435.s007.pdf]

S1 Table: Mass spectrometry data of peptides used in this study

Compound	Chem. Formula<br>Exact mass	Calculated m/z	Measured m/z (ESI)
[Nle^{14},Tyr^{40}-NH_2]Ex-4	$C_{194}H_{293}N_{51}O_{62}$<br>4329.1342	2165.5744 $[M+2H]^{2+}$,<br>1444.0520 $[M+3H]^{3+}$	2165.5986 $[M+2H]^{2+}$<br>1444.0602 $[M+3H]^{3+}$
[Nle^{14},^{127}I-Tyr^{40}-NH_2]Ex-4	$C_{194}H_{292}IN_{51}O_{62}$<br>4455.0309	4456.04 $[M+H]^{+}$	4456.62 $[M+H]^{+}$<br>(MALDI-MS)
[Nle^{14},Tyr^{40}-NH_2]Ex(9-39)	$C_{159}H_{245}N_{41}O_{49}$<br>3512.7940	1757.4043 $[M+2H]^{2+}$<br>1171.9386 $[M+3H]^{3+}$	1757.4082 $[M+2H]^{2+}$<br>1171.9402 $[M+3H]^{3+}$
[Nle^{14},^{127}I-Tyr^{40}-NH_2]Ex(9-39)	$C_{159}H_{244}IN_{41}O_{49}$<br>3638.6906	1820.3526 $[M+2H]^{2+}$<br>1213.9042 $[M+3H]^{3+}$	1820.3547 $[M+2H]^{2+}$<br>1213.9044 $[M+3H]^{3+}$
